# The autophagic inhibition oral squamous cell carcinoma cancer growth of 16-hydroxy-cleroda-3,14-dine-15,16-olide

**DOI:** 10.18632/oncotarget.18987

**Published:** 2017-07-04

**Authors:** Ming-Fang Cheng, Shian-Ren Lin, Fong-Jen Tseng, Yi-Chao Huang, May-Jywan Tsai, Yaw-Syan Fu, Ching-Feng Weng

**Affiliations:** ^1^ Department of Pathology, Tri-Service General Hospital, National Defense Medical Center, Taipei, Taiwan; ^2^ Division of Histological and Clinical Pathology, Hualian Armed Forces General Hospital, Hualien, Taiwan; ^3^ Department of Life Science and Institute of Biotechnology, National Dong Hwa University, Hualien, Taiwan; ^4^ Department of Orthopedics, Hualien Armed Forces General Hospital, Hualien, Taiwan; ^5^ Taoyuan Armed Forces General Hospital, Taoyuan, Taiwan; ^6^ Neural Regeneration Laboratory, Department of Neurosurgery, Neurological Institute, Taipei Veterans General Hospital, Taipei, Taiwan; ^7^ Department of Biomedical Science and Environmental Biology, Kaohsiung Medical University, Kaohsiung, Taiwan

**Keywords:** autophagy, 16-hydroxy-cleroda-3,14-dine-15,16-olide, long-leaf polyalthia, oral squamous carcinoma, xenograft tumor

## Abstract

16-hydroxycleroda-3, 13-dine-15, 16-olide (HCD) isolated from *Polyalthia longifolia* possesses numerous biological activities. Previous studies have reported that HCD can block phosphorylation activity of cancer cells to inhibit tumor cell growth, but the anti-tumor activity in oral squamous cell carcinoma is unrevealed. This study investigates the inhibiting effect of HCD on human OSCC cell growth; thereby, developing a new oral cancer drug. In *in vitro* cultured human OSCC cells (OECM1 and SAS) were employed to test the inhibitory growth of HCD via cell cytotoxic effect using 3-(4, 5-dimethylthiazol-2-yl)-2, 5-diphenyltetrazolium bromide (MTT) assay, Western blotting, and further determining of the inhibitory efficacy of tumor growth by a xenograft tumor on BALB/c male nude mice (*in vivo* test). Under various concentrations of HCD and time course treatments were shown to effectively cause cell death and cell-cycle arrest in OECM1 and SAS cells, which was confirmed via a clinical drug (cisplatin) as a positive control. In addition, HCD induced the autophagic cell death in OECM1 and SAS cells by LC3-mediated LC3-I/LC3-II/p62 pathway at the *in vitro* level. An *in vivo* assay indicated that HCD could treat oral cancer by deferring tumor growth. These findings provide a favorable assessment for further elucidating the role of HCD that targets autophagic cell death pathways as a potential agent for cancer therapy.

## INTRODUCTION

In oral cancer, squamous cell carcinoma, is the most common in both genders, followed by verrucous carcinoma, and then followed the end of the undiffer-entiated carcinoma, small salivary adenocarcinoma. Oral squamous cell carcinoma (OSCC) *in situ* may be confined to the basal layer of the epidermis mucous membrane or the outside of the basal layer only invading the shallow microscopic invasive cancer of the connective tissue, but most human OSCC is diagnosed as one invasive cancer. OSCC is also the most common type of head and neck cancer excluding oropharynx and hypopharynx, the mouth of a narrow definition of classification according to the American Joint Committee on inflammation and the International Union Against Cancer [1]. OSCC is locally destructive, may invade soft tissue and bone, and can be extended to the nerves, lymphatic, and blood vessels throughout the body that results in cervical lymph node metastasis and distant metastasis [2]. In oral cancer, multiple risk factors including foreign carcinogens play an important role. In Taiwan, occurrences of oral cancer are from chewing betel nut, smoking, and drinking; each of these increases the risks for oral cancer according to the relevant literature statistics. When the subject has all three habits, consequently the relative risk of oral cancer increases by 122.8 times [3].

The anti-cancer chemical drugs including 5-FU, cisplatin, paclitaxel, and Ufur are commonly used to treat oral cancer. However, these chemotherapeutic drugs have side effects such as nausea, vomiting, loss of appetite, decreased immunity, oral ulcers, and other adverse effects. Currently, many herbs including Chinese herbs have been applied for OSCC to dampen the aforementioned problems. *Polyalthia longifolia* belongs to the family Annonaceae, is popularly known as “ulta Ashok” in India and widely grown in gardens of tropical and subtropical Asia in the regions of the southern part of Taiwan, Pakistan, and Sri Lanka as an evergreen ornamental tree. *P. longifolia* var. pendula Linn is important in traditional Indian medicine while many part of this tree also have other biological functions [4]. The bark has been reported to have medicinal values to treat skin diseases, fever, hypertension, diabetes, and helminthiasis [5]. A previous study of *P. longifolia* has exhibited anti-inflammatory activity in neutrophils, cytotoxicity towards breast cancer cells, and hepatoma cancer cells [6]. The chemical compounds of *P. longifolia* var. pendula such as diterpenes (clerodane and triterpenes) and aporphine alkaloids have been isolated and investigated for various biological activities. Diterpenoids in the hexane extract of *P. longifolia* seeds shows significant anti-bacterial and anti-fungal activities [7]. Recently, clerodane diterpenes can induce apoptosis of human leukemia HL-60 cells [8]. 16-Hydroxycleroda-3,13-dien-15,16-olide (HCD) and its analogs, extracted from the bark of *P. longifolia* exhibits strong anti-inflammatory activities [9]; enhanced the expression of cyto-protective HO-1 factor and anti-inflammatory enzyme in microglia [10]; the induction of apoptosis in leukemia K562 cells via both a reduction in histone modifying enzymes PRC2-mediated gene silencing and the reactivation of downstream tumor suppressor gene expressions [11] and via PI3K-Akt pathway and Aurora B resulting in gene silencing and cell cycle disturbance [12]. Our previous studies have demonstrated that HCD could cause apoptosis of two CNS cancer cell lines, N18 and C6, via inhibition of FAK-related signaling pathway and accordingly induced the autophagic cell death through ROS generation and p38/ERK1/2 signaling pathway activation [13, 14].

Cisplatin is a traditional anti-cancer agent for treating prostate cancer, bladder cancer, and gastric cancer [15]. Cisplatin has been shown to cause apoptotic cell death [16] and exerts an apoptotic action via mitochondria-mediated activation of caspases [17]. The signals are involved two apoptosome molecules, cytochrome c and Apaf-1, the activation of caspase-9 and caspase-3, downstream molecules leading to mitochondria-mediated apoptosis, and evidenced by cleavage of PARP through the activation of caspase-3. Cisplatin is the most efficient drug used for treating OSCC in clinics. It is applied as a positive control in this study to verify the efficacy of testing compounds. This study investigates whether HCD could result in the inhibition of human OSCC cells (OECM1 and SAS) growth by a MTT assay, flow cytometry assay, and Western blotting. Further study was determined HCD that might cause autophagy through the mTOR/PI3K/Akt/Beclin-1 signaling pathway.

## RESULTS

### Effect of HCD and cisplatin on the cell viability of oral cancer cells

**Figure 1:**
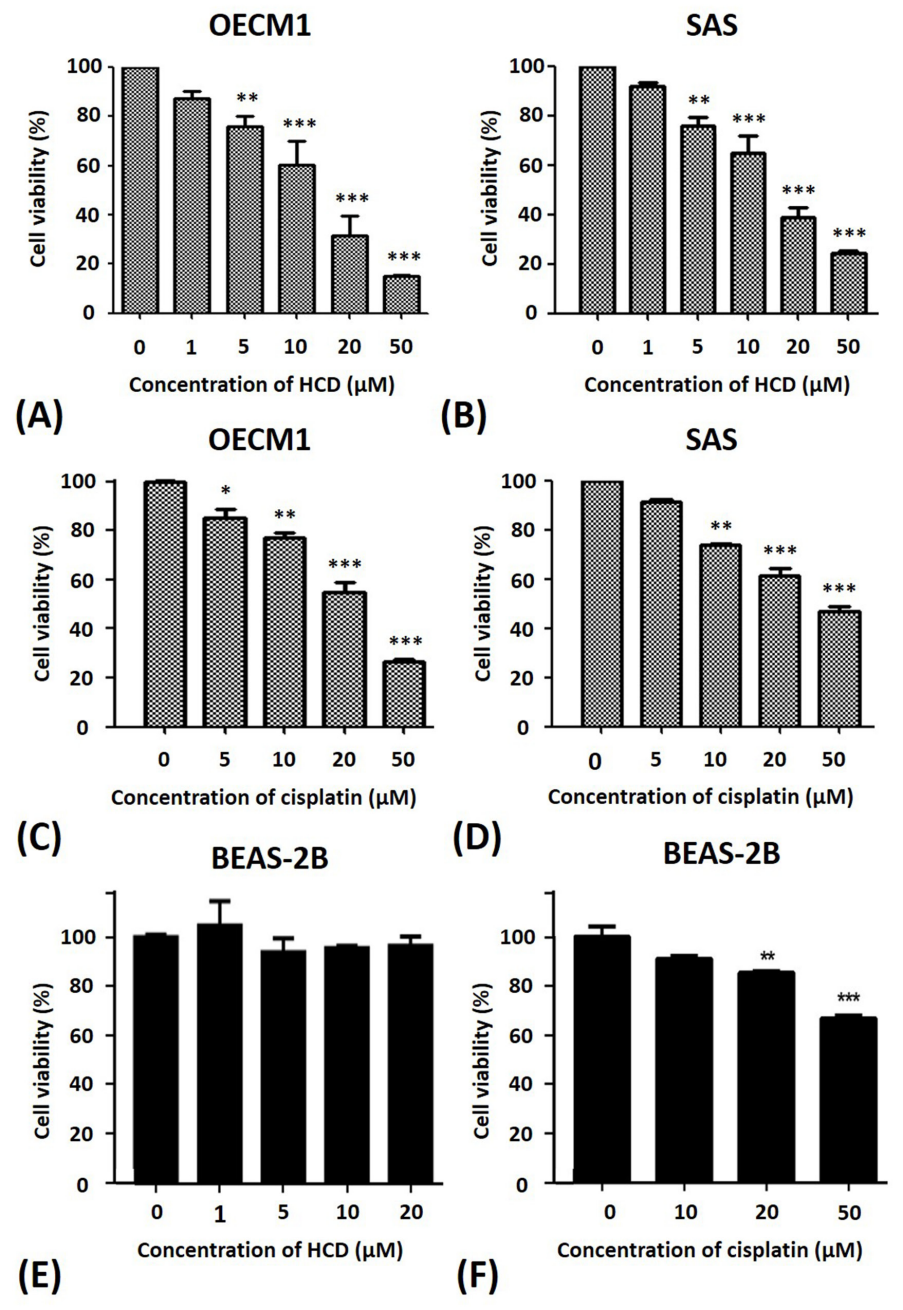
Alterations of the cell viability of OECM1 and SAS cells after HCD and cisplatin treatments **(A)** OECM1, **(B)** SAS, and **(E)** BEAS-2Bcells were incubated with 0 to 50 μM of HCD for 24 h, and the cell viability was determined by a MTT assay. Cell viability of **(C)** OECM1, **(D)** SAS, and **(F)** BEAS-2Bafter cisplatin treatment for 24 h are also shown. The data are presented as the mean ± SE of three independent experiments. * *P* < 0.05, ** *P* < 0.01 and *** *P* < 0.001 when compared with the untreated control (0 μM).

To test the potency of HCD and cisplatin on cell viability of oral cancer cells, OECM1 and SAS cells were incubated with various concentrations of HCD and cisplatin and incubation time courses (24 and 48 h) to measure the cell viability by a MTT assay. The differences of 24 h treated with dose-dependent fashion were observed. Therefore, the 24 hours’ incubation was performed in the subsequent experiments for the efficacy of various concentrations of HCD and cisplatin. Various concentrations (1, 5, 10, 20, and 50 μM) of HCD were employed to treat the cells for 24 h, the data showed that the cell viabilities of both OECM1 and SAS cells were dose-dependent inhibition (*P* < 0.05) in 5, 10, 20, and 50 μM of HCD treatment and IC_50_ of HCD were at the concentrations of 17.79 and 14.79 μM in OECM1 and SAS cells, respectively (Figures 1A & 1B). Additionally, OECM1 and SAS cells were treated with 5, 10, 20, and 50 μM of cisplatin for 24 h, the results indicated that the cell viabilities of both OECM1 and SAS cells were dose-dependent inhibition (*P* < 0.05) of HCD treatment and IC_50_ of cisplatin were at concentrations of 23.44 and 38.91 μM in OECM1 and SAS cells, respectively (Figures 1C & 1D). Normal BEAS-2B cells were incubated with 1, 5, 10, and 20 μM of HCD for 24 h (Figure 1E), there was no effect cell viability of BEAS-2B cells at various concentrations of HCD treatment. While the BEAS-2B cells were treated with 10, 20, and 50 μM of cisplatin for 24 h, the data revealed that the IC_50_ of cisplatin at the concentration of 97.45 μM (Figure 1F). Taken together, these results demonstrated that HCD is non-cytotoxic to the normal cells and the potency of HCD for the suppressing growth of OECM1 and SAS cells is close to the cisplatin.

### Effect of HCD and cisplatin on the cell cycle of oral cancer cells

To determine the phase of cell cycle in HCD and cisplatin treatments, OECM1 and SAS cells were incubated with various concentrations of HCD and cisplatin, and stained with propidium iodide followed by flow cytometry. OECM1 cells had significantly increased in the G_0_/G_1_ phase (49.6 ± 0.1% to 50.5 ± 0.2%, *P* < 0.05) when treated with 1, 5, and 10 μM of HCD at 12 h, but no significant difference at 24 h incubation was found (Table 1). In addition, OECM1 cells treated with 1, 5, and 10 μM of HCD for 12 h had higher percentage of G_0_/G_1_ phase than those of HCD treatment for 24 h. When SAS cells treated with 1, 5, and 10 μM of HCD for 12 and 24 h, 24 h HCD-treated cells were significantly increased in G_0_/G_1_ phase (37.1 ± 0.4% to 43.8 ± 1.4%, *P* < 0.05) (Table 2). The sub-G_1_ phase was increased from 1.9 ± 0.3% to 3.3 ± 0.4% in SAS cells treated with 10 μM of HCD for 24 h (*P* < 0.05). Moreover, the sub-G_1_ phase was increased from 1.3 ± 0.2% to 29.0 ± 4.5% of OECM1 cells with 20 μM of cisplatin treatment for 24 h (*P <* 0.05) (Table 3). The sub-G_1_ phase was increased from 1.5 ± 0.3% to 18.4 ± 0.3% of SAS cells with 20 μM of cisplatin treatment for 24 h (*P <* 0.05). In addition, with 20 μM cisplatin treatment for 12 h did not show the increase sub-G_1_ or G_0_/G_1_ phase in OECM-1 and SAS cells (Table 4). When compared to the cell apoptosis of cisplatin treatment, these results revealed that HCD could induce cell death through autophagy in OECM1 and SAS cells.

**Table 1:** HCD mediated the cell cycle distribution in OECM1 cells

Cell	Dosage (μM)	Sub G1 (%)	G0/G1 (%)	S (%)	G2/M (%)
**OECM1**	0	0.6±0.1	49.6±0.1	17.2±0.6	32.6±0.2
**12 h**	1	1.1±0.2	48.2±0.4	18.9±1.8	31.8±0.7
	5	1.4±0.7	46.8±0.4	19.3±1.4	32.5±2.9
	10	0.9±0.4	50.5±0.2**	17.1±3.0	31.4±1.5
**24 h**	0	1.3±0.2	48.4±1.3	14.5±2.6	35.8±1.2
	1	1.3±0.2	47.4±2.7	14.4±2.5	37.0±0.5
	5	1.5±0.5	46.1±4.0	15.8±3.6	36.6±0.2
	10	2.2±1.1	38.1±3.5	13.7±0.5	46.0±1.4***

OECM1 cells were treated with 1, 5, and 10 μM of HCD for 12 and 24 h, respectively and stained with propidium iodide. The results are presented as the mean ± SE of three independent experiments. ** *P* < 0.01 and *** *P* < 0.001 when compared with the untreated control (0 μM).

**Table 2:** HCD mediated the cell cycle distribution in SAS cells

Cell	Dosage (μM)	Sub G1 (%)	G0/G1 (%)	S (%)	G2/M (%)
**SAS**	0	1.9±0.7	37.0±2.2	28.1±3.7	33.0±2.4
**12 h**	1	1.7±0.3	35.3±3.5	28.1±3.5	34.8±2.1
	5	0.8±0.3	39.6±1.2	23.8±3.1	35.7±2.3
	10	1.3±0.5	39.2±0.8	21.6±3.2	37.9±1.1
**24 h**	0	1.9±0.3	37.1±0.4	24.4±1.2	36.6±3.0
	1	2.2±0.1	37.5±1.6	21.4±4.7	39.0±2.7
	5	2.2±0.2	36.8±0.4	27.4±1.0	33.6±3.4
	10	3.3±0.4**	43.8±1.4***	24.8±0.3	28.0±1.9

SAS cells were treated with 1, 5, and 10 μM of HCD for 12 and 24 h, respectively and stained with propidium iodide. The results are shown as the mean ± S.E. of three independent experiments. ** *P* < 0.01 and *** *P* < 0.001, when compared with the untreated control (0 μM).

**Table 3:** Cisplatin mediated the cell cycle distribution in OECM1 cells

Cell	Dosage (μM)	Sub G1 (%)	G0/G1 (%)	S (%)	G2/M (%)
**OECM1**	0	0.8±0.2	48.0±0.5	19.7±2.4	31.5±0.3
**12 h**	5	1.3±0.5	41.3±1.9	24.0±0.9	33.5±0.5
	10	0.6±0.1	38.8±0.4	28.7±0.9	32.0±2.1
	20	0.7±0.1	46.9±0.3	26.0±3.5	26.4±0.9
**24 h**	0	1.3±0.2	45.0±3.8	21.3±2.1	32.4±3.5
	5	2.3±0.4	34.6±4.7	41.4±5.7^**^	21.7±2.6
	10	9.4±2.1	47.8±2.9	25.6±2.1	17.2±3.8^*^
	20	29.0±4.5^***^	34.2±4.4	18.4±0.4	18.4±4.9^*^

OECM1 cells were treated with 5, 10, and 20 μM of Cisplatin for 12 and 24 h, respectively and stained with propidium iodide. The results are shown as the mean ± S.E. of three independent experiments. * *P* < 0.05, ** *P* < 0.01 and *** *P* < 0.001, compared with the untreated control (0 μM).

**Table 4:** Cisplatin mediated the cell cycle distribution in SAS cells

Cell	Dosage (μM)	Sub G1 (%)	G0/G1 (%)	S (%)	G2/M (%)
**SAS**	0	1.3±0.2	39.3±1.7	24.3±3.8	35.1±1.3
**12 h**	5	1.1±0.1	35.7±4.1	28.5±2.2	34.7±0.8
	10	1.0±0.1	43.2±3.7	24.8±6.2	31.0±1.8
	20	2.5±1.1	51.6±1.4	22.0±6.6	23.9±2.6
**24 h**	0	1.5±0.3	41.3±6.4	25.8±2.9	31.4±5.1
	5	5.1±2.3	59.0±0.9^*^	22.0±5.1	13.9±2.8^**^
	10	9.7±0.9^*^	54.9±2.9	17.6±4.6	17.7±0.6^*^
	20	18.4±3.1^***^	44.8±4.1	16.7±2.8	20.0±1.1

SAS cells were treated with 5, 10, and 20 μM of Cisplatin for 12 and 24 h, respectively and stained with propidium iodide. The results are shown as the mean ± S.E. of three independent experiments. * *P* < 0.05, ** *P* < 0.01 and *** *P* < 0.001, when compared with the untreated control (0 μM).

### Effects of HCD on the mTOR protein levels of the autophagy in oral cancer cells

**Figure 2:**
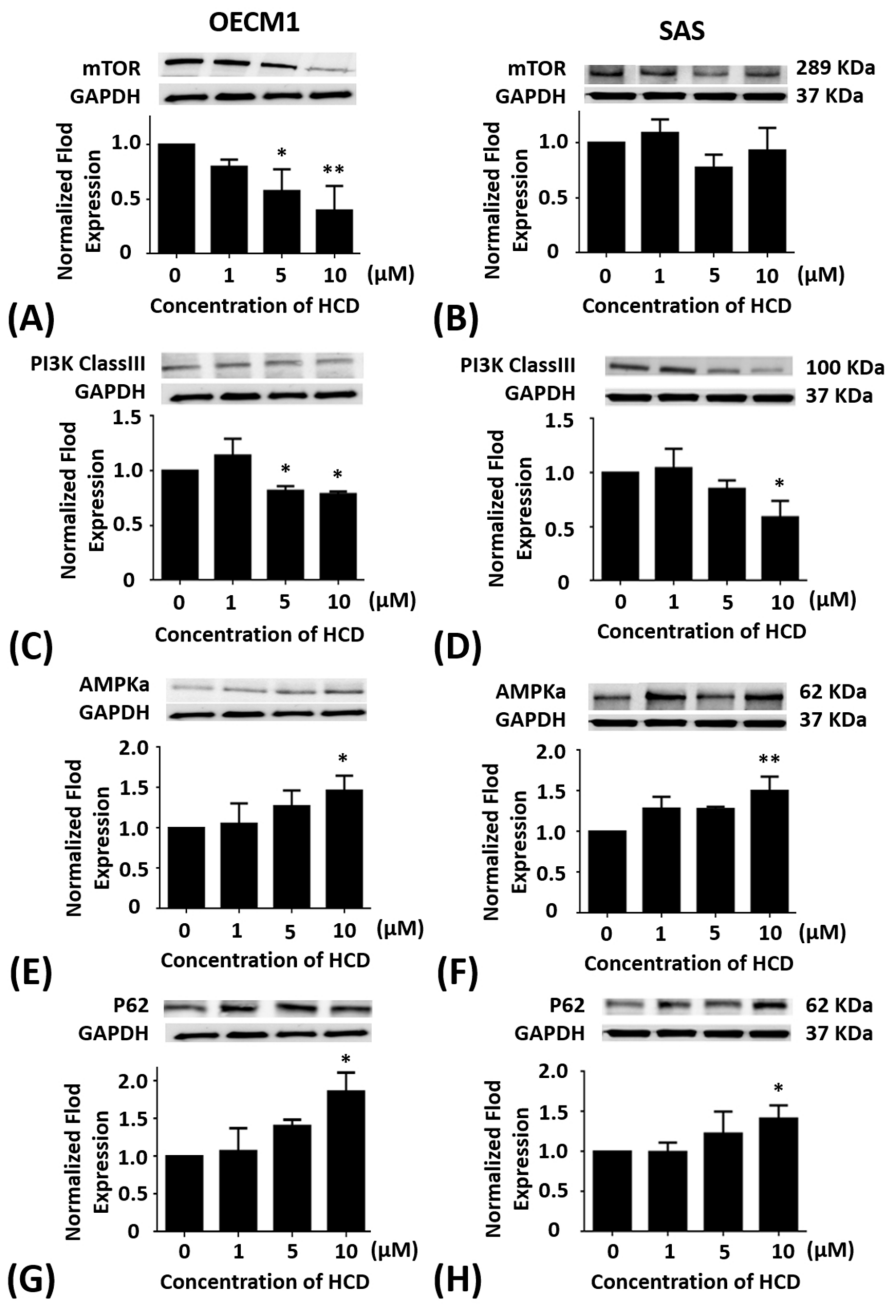
Altered protein levels of mTOR, PI3K Class III, AMPKα, and P62 of OECM1 and SAS cells treated with HCD OECM1 and SAS cells were treated with 1, 5, and 10 μM of HCD for 24 h. Cells were lysed in RIPA buffer for the Western blot. Quantization of the expressions of **(A** and **B)** mTOR, **(C** and **D)** PI3K Class III, **(E** and **F)** AMPKα, and **(G** and **H)** P62 protein levels were performed, respectively. The results were presented as the mean ± SE of three independent experiments. *P < 0.05 and ** *P* < 0.01 when compared with the untreated control (0 μM).

OECM1 and SAS cells were treated with 1, 5, and 10 μM of HCD for 24 h, respectively. In OECM1 cells, the protein levels of mTOR had a significant (*P <* 0.05) decrease in 5 and 10 μM of HCD treatments when compared with the untreated control (0 μM) (Figure 2A). When compared with the untreated control, the protein levels of mTOR in SAS cells were no effect of HCD treatments (Figure 2B).

### Effects of HCD on the PI3K-classIII protein levels of the autophagy in oral cancer cells

OECM1 and SAS cells were treated with 1, 5, and 10 μM of HCD for 24 h, respectively. In OECM1 cells, the protein levels of PI3K-ClassIII had significant (*P <* 0.05) decrease in 5 and 10 μM of HCD treatments when compared with the untreated control (Figure 2C). When compared with the untreated control, the protein levels of PI3K-ClassIII in SAS cells were significantly (*P <* 0.05) down-regulated in 10 μM of HCD treatment (Figure 2D).

### Effects of HCD on the AMPKα protein levels of the autophagy in oral cancer cells

OECM1 and SAS cells were treated with 1, 5, and 10 μM of HCD for 24 h, respectively. In OECM1 cells, the protein levels of AMPKα had significant (*P <* 0.05) increase in 1, 5 and 10 μM of HCD treatments when compared with the untreated control (Figure 2E). In SAS cells, the protein levels of AMPKα were also elevated in 1, 5, and 10 μM of HCD treatments when compared with the untreated control (*P <* 0.05) (Figure 2F).

### Effects of HCD on the P62 protein levels of the autophagy in oral cancer cells

OECM1 and SAS cells were treated with 1, 5, and 10 μM of HCD for 24 h, respectively. In OECM1 cells, the protein levels of P62 had significant (*P <* 0.05) increase in 5 and 10 μM of HCD treatments when compared with the untreated control (Figure 2G). In SAS cells, the protein levels of P62 had significant (*P <* 0.05) increase in 10 μM of HCD treatments when compared with the untreated control (Figure 2H).

### Effects of HCD on the Akt protein levels of the autophagy in oral cells

**Figure 3:**
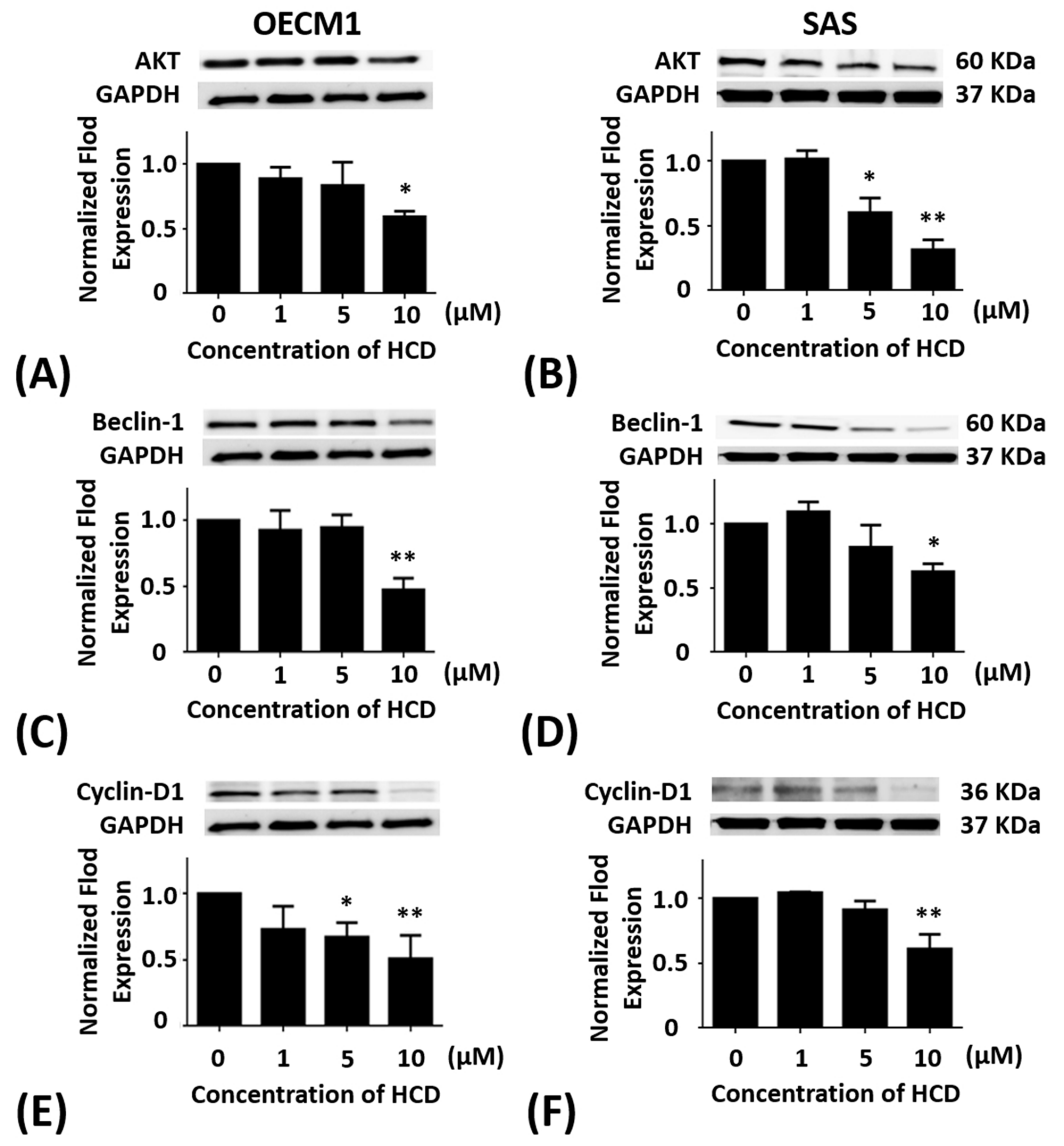
Altered protein levels of Akt, Beclin-1, and cyclin D1 of OECM1 and SAS cells treated with HCD OECM1 and SAS cells were treated with 1, 5, and 10 μM of HCD for 24 h. Cells were lysed in RIPA buffer for Western blot. Quantization of the expressions of **(A** and **B)** Akt, **(C** and **D)** Beclin-1, and **(E** and **F)** cyclin D1 protein levels were performed, respectively. The results were presented as the mean ± SE of three independent experiments. *P < 0.05 and ** *P* < 0.01 when compared with the untreated control (0 μM).

OECM1 and SAS cells were treated with 1, 5, and 10 μM of HCD for 24 h, respectively. In OECM1 cells, the protein levels of Akt had significant (*P <* 0.05) decrease in 10 μM of HCD treatments when compared with the untreated control (Figure 3A). When compared with the untreated control, the protein levels of Akt in SAS cells were significantly (*P <* 0.05) decreased in 5 and 10 μM of HCD treatments (Figure 3B).

### Effects of HCD on the beclin-1 protein levels of the autophagy in oral cancer cells

OECM1 and SAS cells were treated with 1, 5, and 10 μM of HCD for 24 h, respectively. In OECM1 cells, the protein levels of Beclin-1 had significant (*P <* 0.05) decrease in 10 μM of HCD treatments when compared with the untreated control (Figure 3C). In SAS cells, the protein levels of Beclin-1 were significantly decreased in 5 and 10 μM of HCD treatments when compared with the untreated control (*P <* 0.05) (Figure 3D).

### Effects of HCD on the cyclin D protein levels of the autophagy in oral cancer cells

OECM1 and SAS cells were treated with 1, 5, and 10 μM of HCD for 24 h, respectively. In OECM1 cells, the protein levels of cyclin D had significant decrease in 5 and 10 μM of HCD treatments when compared with the untreated control (*P <* 0.05) (Figure 3E). In SAS cells, the protein levels of cyclin D had significant decrease in 10 μM of HCD treatments when compared with the untreated control (*P <* 0.05) (Figure 3F).

### Effects of HCD on the LC3-I & II protein levels of the autophagy in oral cancer cells

**Figure 4:**
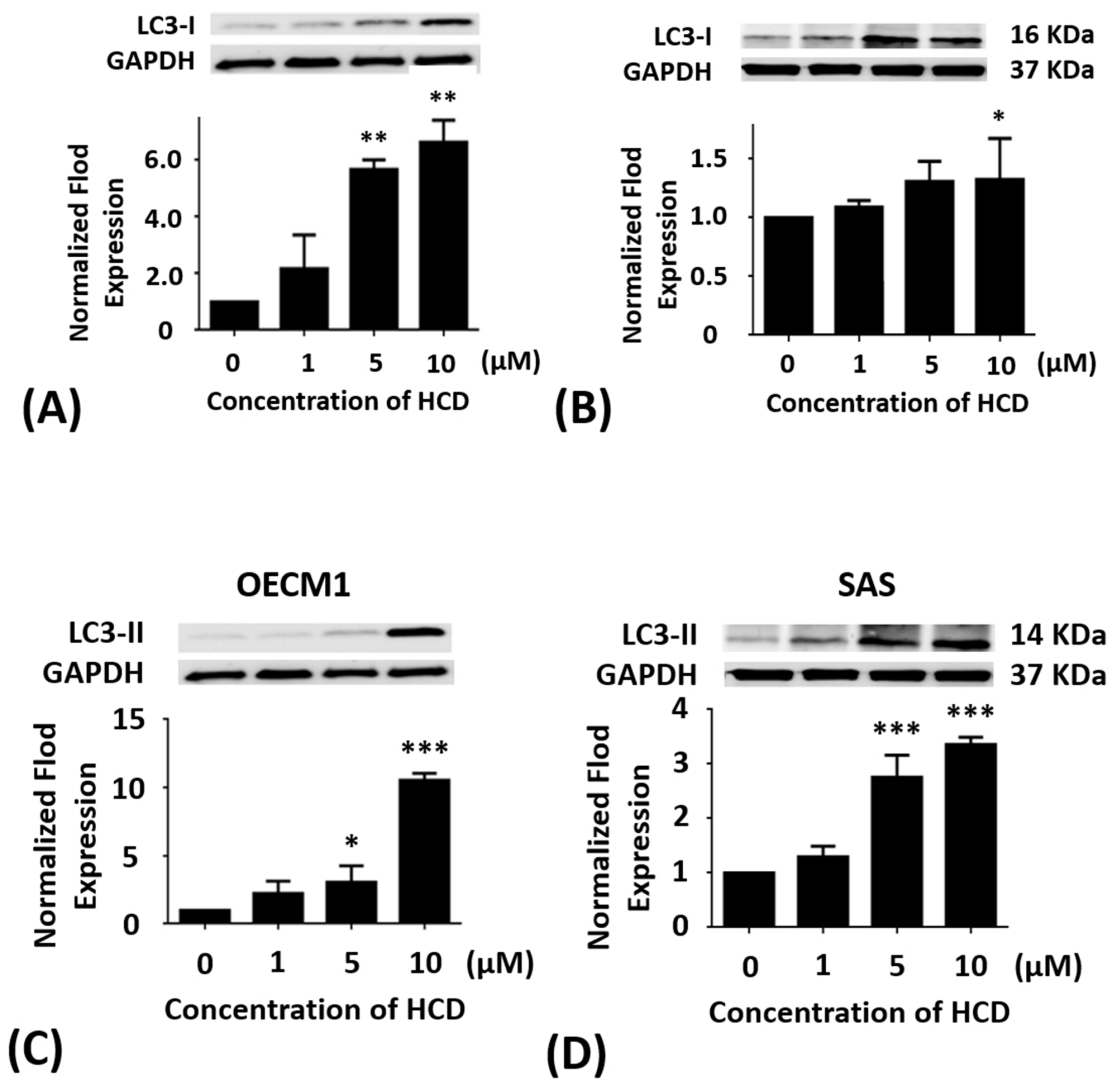
Altered protein levels of LC3-I and II of OECM1 and SAS cells treated with HCD OECM1 and SAS cells were treated with 1, 5, and 10 μM of HCD for 24 h. Cells were lysed in RIPA buffer for the Western blot. Quantization of the expressions of **(A** and **B)** LC3-I and **(C** and **D)** LC3-II protein levels were performed, respectively. The results were presented as the mean ± SE of three independent experiments. *P < 0.05, **P < 0.01 and ***P < 0.001 when compared with the untreated control (0 μM).

OECM1 and SAS cells were treated with 1, 5, and 10 μM of HCD for 24 h, respectively. In OECM1 cells, the protein levels of LC3-I & II had significant (*P <* 0.05) increase in 5 and 10 μM of HCD treatments when compared with the untreated control. In SAS cells, the protein levels of LC3-I & II had significant (*P <* 0.05) increase in 10 μM of HCD treatments when compared with the untreated control (Figure 4). From all Western blots data demonstrated that HCD indeed caused cell death via autophagy in OECM1 and SAS cells.

### Suppressive effects of HCD on the tumor of SAS cells-bearing xenograft mice

**Figure 5:**
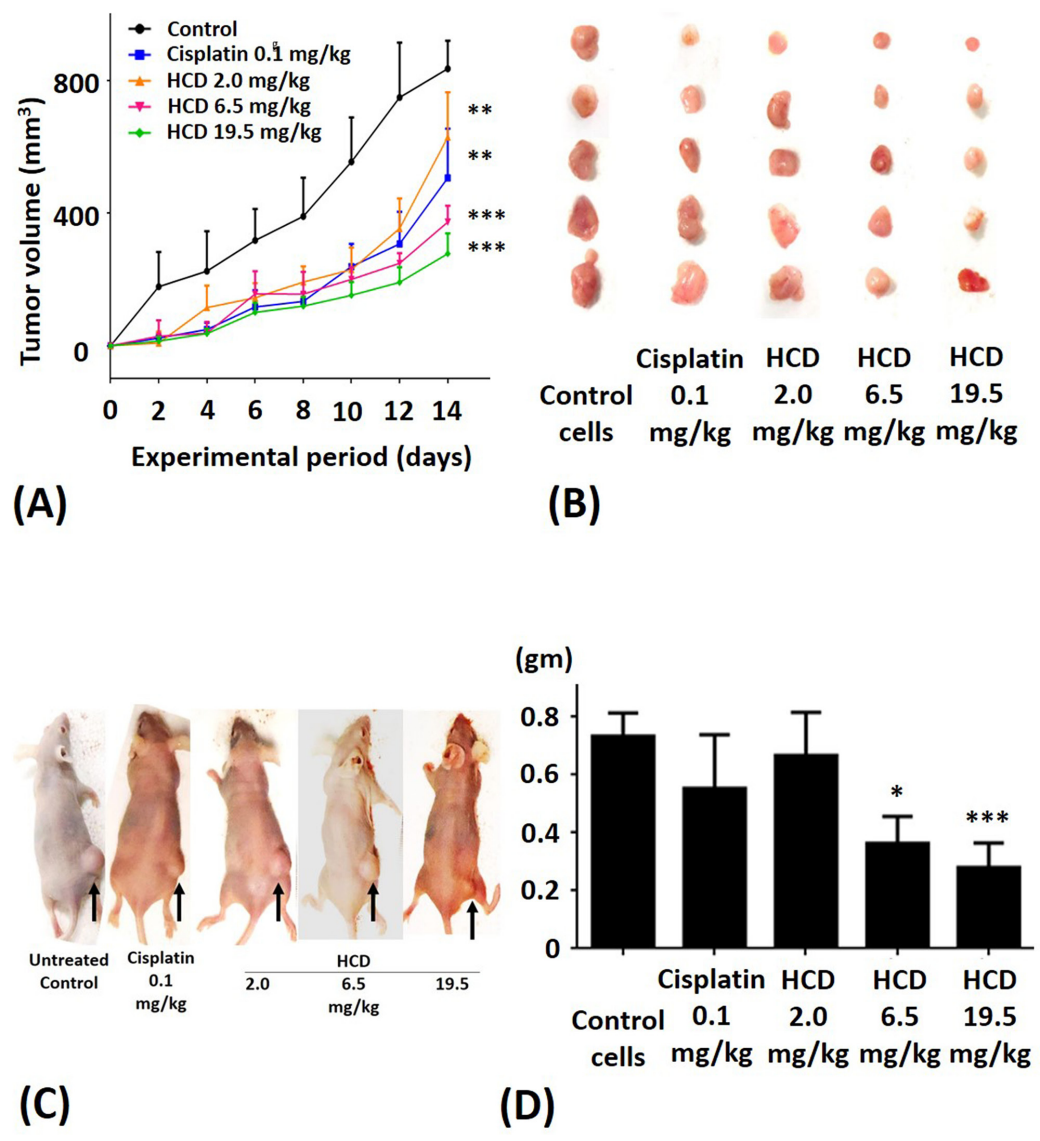
The xenograft tumor growth of SAS after treating with HCD and cisplatin SAS cells were injected in nude mice and treated with 2.0, 6.5, and 19.5 mg/kg B.wt of HCD or 0.1 mg/kg B.wt of cisplatin. Tumor growth was observed via **(A** and **B)** tumor volume, **(C)** macroscopic observation, and **(D)** tumor weight. The results are presented as the mean ± SE of six mice. *P < 0.05, **P < 0.01 and ***P < 0.001 when compared with the untreated control (0 μM).

To validate anti-tumor efficacy of HCD on the SAS cells-bearing xenograft mice, various dosages of HCD and cisplatin (reference drug as a positive control) were employed to test in SAS cells-xenograft mice. We also monitored the body weight of nude mice during HCD and cisplatin treatments and the result showed no significant change among the control and treated groups (data not shown). The tumor volume of SAS cells xenograft mice was significantly affected by HCD treatment (Figure 5A). After treatment for 2 weeks, the average tumor sizes were 629, 373, and 277 mm^3^ in the 2.0, 6.5, and 19.5 mg/kg B.wt of HCD treated groups, respectively. The average tumor size was 506 mm^3^ in the 0.1 mg/kg cisplatin treated group. However, the average tumor size was 836 mm^3^ in the control group. The rates of inhibition by HCD treatment were 24.8, 55.4, and 66.9% in the 2.0, 6.5, and 19.5 mg/kg B.wt of HCD treated groups, respectively; and was 39.4% in 0.1 mg/kg cisplatin treated group.

**Figure 6:**
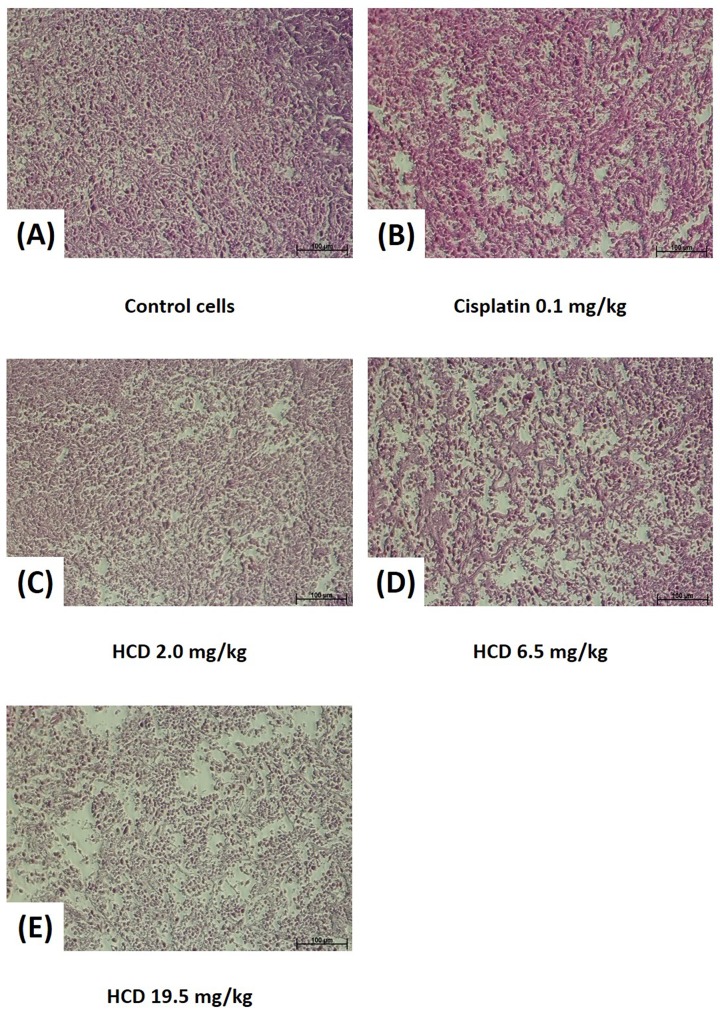
Histopathology of SAS xenograft tumor after treating with HCD and cisplatin Xenograft tumor of SAS cells after treating with **(B)** 0.1 mg/kg B.wt cisplatin or **(C)** 2.0, **(D)** 6.5, and **(E)** 19.5 mg/kg B.wt of HCD were sliced and stained with hematoxylin and eosin. Tumor structure was observed and compared with the **(A)** untreated control (0 μM).

The macroscopic appearance of tumor in xenograft nude mice had smaller tumor weight when treated with 2.0, 6.5, and 19.5 mg/kg B.wt of HCD as compared with the untreated control group (Figure 5B & 5C). The tumor volume of nude mice had significantly decreased in 2.0, 6.5, and 19.5 mg/kg B.wt of HCD treated groups when compared with the untreated control group (*P <* 0.05) (Figure 5D). The histological changes in tumor tissues were assessed by H&E staining to determine the tumor cell arrangement density. The cell arrangements of control group were denser than those of HCD treated groups (Figure 6). These results revealed that HCD suppressed tumor growth in SAS cells-bearing nude mice, suggesting long-leaf *Polyalthia* possesses the potential for anti-tumor efficacy in human oral squamous cell cancer.

## DISCUSSION

HCD was obtained from *P. longifolia* and induced differential consequences from an *in vitro* assay such as cell toxicity, cell cycle, and autophagy signaling pathways in different types of OSCC cell lines (OECM1 and SAS). In BEAS-2B normal cell treated with various concentrations of HCD had no effect on cell viability, which indicated that HCD is non-cytotoxic (Figure 1E). When compared to the cytotoxicity of cisplatin in OECM1 and SAS cells with IC_50_ values of 23.44 and 38.91 μM, respectively (Figure 1C&1D); HCD had moderate IC_50_ values of 17.79 and 14.79 μM for cell viability, respectively (Figure 1A&1B); and induced OECM1 and SAS cell death with a dose-dependent manner. Increases in G_0_/G_1_ phase cell cycle arrest with increasing doses of HCD treatments in OECM1 and SAS cells were apparent (Tables 1 & 2). There was no increase of cells in the sub-G_1_ phase with various doses of HCD. The results also showed that HCD activated the protein levels of LC3-I & II (Figure 4). And also, it is well documented that LC3-I and LC3-II both proteins are involved in the development of autophagy and play an important role during autophagy [18]. Notably, autophagy can have two opposing effects, namely cytoprotection and autophagic cell death [19]. Autophagy is a catabolic pathway used by cells to support metabolism in response to starvation and to clear damaged proteins and organelles in response to stress [20]. Moreover, autophagy has been reported to play roles in anticancer therapy in multiple cancers [21]. In the present results showed that HCD has an inhibitory role on oral cancer cells partially via the induction of cell cycle arrest and cell death by autophagy.

In this study, clinical drug cisplatin, as a reference control, exerts its apoptotic action by the mitochondria-mediated activation of caspases [16]. Moreover, the mechanism of cisplatin action on apoptosis cell death has been elucidated [17]. Apoptosis has two apoptosome molecules, cytochrome c and Apaf-1, the activation of caspase-9 and caspase-3, downstream molecules leading to mitochondria-mediated apoptosis, and is evidenced by the cleavage of PARP through the activation of caspase-3 and were detected after cisplatin-treated. In both Tables 3 & 4 show that treatment with 20 μM of cisplatin could remarkably induce higher sub-G_1_ phase cells in OECM1 and SAS cells, revealing that cisplatin-treated lead to cell death by apoptosis [15, 22]. This further confirmed that HCD treatments did not affect any dramatic sub-G_1_ change, implying that the cell death of HCD-treated in OECM1 cells might be through different pathways via a non-apoptotic fashion. While SAS cells treated with HCD showed cell cycle arrest represented as cell autophagy, suggesting the cause of HCD on cell death of SAS is through signal pathway via an autophagic manner.

OECM1 is a non-tumorigenic human gingival squamous carcinoma cell line with a p53 missense mutation at codon 173 [23]. Unlike OECM1, SAS, a tongue carcinoma cell line, is a tumorigenic cell line with wild-type p53 activity [24]. In researching OSCC chemotherapy, cucurbitacin E, a natural compound from family Cucurbitaceae, could be a potent chemo-preventive agent against OSCC [18]. Andrographolide, extracted from *Andrographis paniculata*, could enhance radio-sensitivity in oral cancer [25]. Again, curcumin could decrease DNA adducts, induce NF-κB activity, and delay tumorigenesis in oral cancer cells [26]. Recently, OECM1 and SAS cells were triggered via the autophagic pathway with the mammalian target of rapamycin (mTOR)/phosphatidylinositol 3 kinase (PI3K)/Akt/Atg6 (Beclin-1)/p70 ribosomal protein S6 kinase (p70S6K) signal pathways after tetrandrine treatment [18]. The Ras/RAF/MEK/ERK signaling pathways activated the proliferation and death of cancer cells are frequently associated with the induction of autophagy [27]. Of note, autophagy is a catabolic process of the eukaryotic cells [28] and has been extensively studied in cancer therapy. However, the results of cell cycle analysis showed no sub-G_1_ increase in two cell lines after HCD treatment, the upstream apoptosis signaling pathway cannot be excluded that the interaction of autophagy and apoptosis is frequently observed. Beclin-1, p53, caspase-3, 6, and 9 are the key signal transducer in crosstalk of autophagy/apoptosis which p53, capase-3, 6, and 9 can act as down-regulators and Beclin-1 as an up-regulator [29]. Four stages of autophagic formation including initiation, nucleation, elongation, and maturation have been classified, Beclin-1 in nucleation stage as a critical role is activated by ULK-1 and forms protein complex with Vps34 and Vps15 proteins, which triggers formation of LC3 proteins and autophagosome [30]. In addition, Beclin-1-independent signal pathway is associated with PI3K-ClassIII and induces p62 activity [31], suggesting that Beclin-1-independent signal pathway is also involved in the activation of autophagy. Thereby the involvement Beclin-1-dependent and -independent pathways in the induction of autophagy is taken into consideration while HCD treatment resulted in the autophagy of OSCC cells with Beclin-1-dependent pathway. This issue is discussed in following section.

In the present study, the autophagic inhibition of HCD in two cell lines may act on different signal pathways. In OECM1 cells, mTOR, Beclin-1, and PI3K-ClassIII are decreased and AMPK is increased, while Beclin-1 and PI3K-ClassIII are decreased and AMPK is increased without change mTOR in SAS cells. There are two pathways, which are well known to regulate the autophagy. In response to starvation in mammalian cells: the class I PI3KClass-III/Akt/mTOR/p70S6K signaling pathway and the Ras/Raf/MEK/ERK1/2 pathway [32]. PI3K class III is activated through IGFR-related signaling pathway and relays its signal to Akt [33]. Subsequently, Akt stimulates a central regulator of autophagy, mTOR. As diverse stimuli including energy deprivation presence, activities of mTOR, and AMP activated protein kinase (AMPK) would be modulated and consequently activated autophagy [34]. Western blot results showed HCD-induced autophagy in SAS cell was associated with the involvement of the mTOR/PI3K-ClassIII/P62/Beclin-1/cyclin D signaling pathways (Figure 2B, 2D, and 2H; 3D and 3F). While the results showed HCD-induced autophagy in OECM1 cell was associated with the involvement of the mTOR/AMPKα/Akt/Beclin-1/cyclin D/LC3-I/LC3-II signaling pathways (Figure 2A and 2E; 3A, 3C, and 3E; 4A and 4C). On the other hand, the Ras/Raf/ERK pathway is among the most commonly deregulated pathways identified in tumors, as indicated by frequently observed activating mutations in Ras-mediated activation of Raf-Mek1 and ERK oncogenes [35]. Activated Ras-expressing cells are dependent on autophagy to survive during starvation [36]. In the most of the studies related to cell death, it was induced by the Ras/Raf/ERK pathway and ERK activation is unusually prolonged [37]. The prolonged ERK activation has been shown to promote the death of human cancer cell lines from different origins and this property of the Ras/Raf/ERK pathway to induce cell death could be used to target cancer cells [38]. ERK has also been shown to induce autophagy in response to a number of anti-tumor/cytotoxic agents, such as capsaicin in breast cancer cells and cadmium in mesangial cells [39]. According to the alterations of Ras/Raf/ERK pathway followed HCD incubation is not investigated in the present study and needs to be further explored. The important functions of Akt are associated with cell survival while Akt might suppress autophagy and apoptosis. The activation of Akt has been proposed to be a mechanism of autophagy suppression based on several observations [40]. The inhibition of Akt is associated with an increase mitochondrial superoxide and cellular reactive oxygen species (ROS) levels to cause the induction of apoptosis and autophagy [41]. Our findings indicate that the decrease of mTOR, PI3 kinase class III, Akt, and cyclin D levels are involved in cell death by HCD in OECM1 cells supports by previous results [41]. One study has been reported that Akt expression could down-regulate LC3-II expression, thereby inhibiting the autophagy [42]. The Akt/mTOR pathway suppresses the autophagy, whereas the ERK1/2 pathway positively potentiates the autophagy. These signaling pathways are often activated in numerous types of tumors and are associated with tumorigenesis. Therefore, in cancer cells, these signaling pathways likely play a significant role in regulating autophagy as well as in oncogenesis [43]. In this study revealed that HCD induced autophagy of OECM1 and SAS cells by LC3-mediated LC3-I/LC3-II/p62 pathway and consequently lead to the autophagic cell death.

Nowadays, xenograft mice model is an important approach in research of tumor biology and pharmacology. There are two main xenografts such as orthotopic and heterotopic injection/transplantation to meet those investigations demand. Thereby the experimental detail, especially the site of tumor transplantation, is challenged that it is directly related to reliability. Notably, orthotopic xenograft is more precise for examining the morphology of tumor growth and formation in situ to respond the accomplishment of therapy or treatment in exact cancer of patient. Conversely, the potentials of heterotopic transplantation such as easy handle and monitoring, immunocompetent host and non-immunogenic, and strong database support. However, there may have some limitations of heterotopic xenograft including (1) Heterotopic injection is lack of tumor/stroma interaction [44]. (2) In comparison with the microenvironment of orthotopic transplantation, local invasion and metastasis is less observed in heterotopic transplantation [45]. Obviously, heterotopic xenograft is easier tracing the tumor growth via Vernier scale to measure the size and volume of tumor without image monitor. Currently, this heterotopic xenograft is accepted and extensively performed by researchers and physicians for the drug discovery in cancer therapy. Heterologous with heterotopic xenograft in this study was executed as an animal model for the evaluation of anti-tumor efficacy of HCD. After HCD treatment resulted in shrinkage of tumor volume and size in heterotopic inoculated mice (Figure 5) possible due to tumor cell death, implying that HCD inhibited tumor growth. Nonetheless, recurrence is one critical issue in cancer treatment because the prognosis is associated with recurrent frequency. It has been elucidated that ω-6/ω-3 fatty acid ratio in diet after surgery can modulate the recurrence of prostate cancer transplanted mice [46]. Moreover, CD-133-targeted nanoparticles conjugated with paclitaxel can inhibit xenograft tumor recurrence [47]. In our preliminary data revealed that the tumors of mice were recurrent when withdraw HCD treatment. Interestingly, the suppression of tumor size in tumor-mouse retreated with HCD was still observed (data not shown). The clinical application of HCD needs to be further elucidated.

Recently, HCD exhibits anti-cancer effect on neuroblastoma, glioma, and oral squamous cell lines, the application is still a challenge due to its low water solubility [13, 14]. In order to amend this disadvantage, numerous novel approaches have been investigated such as nano-encapsulation. Nano-scale carriers have been extensively evaluated for promoting abovementioned problem and mesoporous silica nanoparticles (MSNs), one of nano-carrier can load/release drug directly via its pore channels and target to specific cells by functional surface [48]. Through these features, smart and biocompatible drug delivery system can be feasible for medical use such as chemotherapeutic drugs [49]. In solicitation of MSN-conjugated drug delivery system, various therapeutic purposes such as cancer [50–54], ischemia [55], bacterial infections [56], others such as imaging [57, 58], and biomedical applications [59] have been elucidated. Remarkably, the applications of MSN conjugated with natural compounds such as quercetin in breast cancer [60], curcumin in breast cancer and colon cancer [61, 62], and resveratrol in colon cancer [63] have been tested in enhancement of bioavailability and solubility. These results are associated with MSN-HCD for upgrading HCD’s potential and promise in clinical therapy [64].

**Figure 7:**
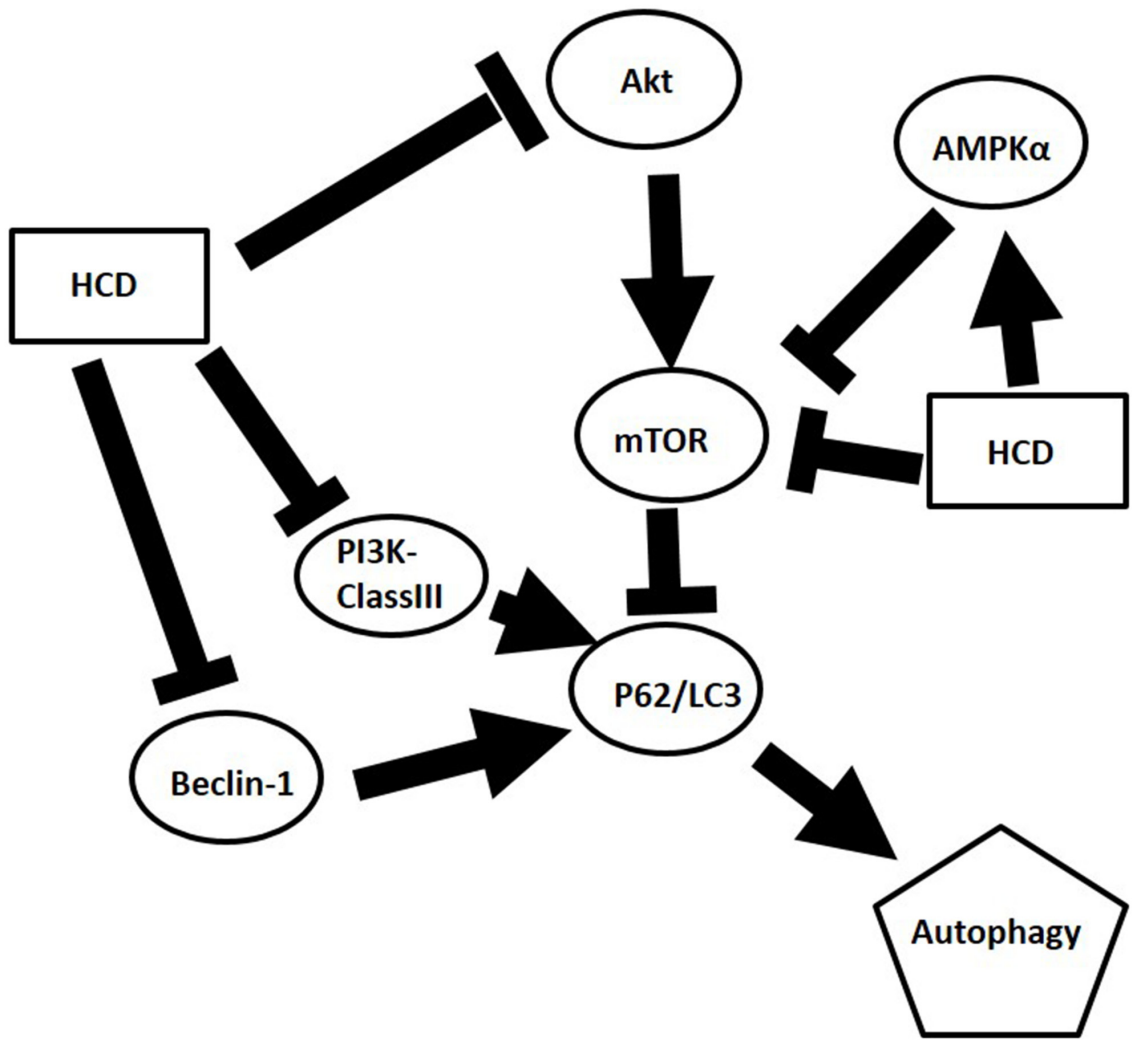
Graphic abstract of postulated mechanism of HCD in OSCC

In conclusion, the present results showed HCD could induce autophagic cell death through inhibiting mTOR and PI3K-classIII/Beclin-1 related signaling and activating AMPKα/P62/LC3-I/II signaling pathway in OSCC cells (Figure 7) but not in the normal cells-BEAS-2B cell line. Moreover, this autophagic cell death via HCD also inhibited xenograft OSCC tumor growth in nude mice. These results provide promising evidence that HCD has potential as a chemotherapeutic reagent for squamous cell carcinoma.

## MATERIALS AND METHODS

### 16-Hydroxycleroda-3,13-dien-15,16-olide (HCD) and cisplatin

16-Hydroxycleroda-3,13-dien-15,16-olide (HCD) was extracted from the bark of *P. longifolia* as briefly described in a previous study [13]. (*SP*-4-2)- diamminedichloroplatinum (II) (cisplatin, TEVA Pharma B.V., Haarlem, Netherlands) was taken as a positive control in this study.

### Cell culture

Human gingival squamous carcinoma cell line OECM1 and tongue carcinoma cell line SAS were obtained from Dr. Ta-Chun Yuan (Department of Life Sciences, National Dong-Hwa University, Hualien, Taiwan) and human bronchus epithelial cell BEAS-2B was obtained from Dr. Chih-Jen Yang (Department of Internal Medicine, Kaoshiung Medical University Hospital, Kaoshiung, Taiwan). All assays were carried out within 20 passages to ensure a uniform cell population and reproducibility.

OECM1 was cultured in RPMI 1640 medium (Thermo-Fisher, Waltham, USA) supplemented with 5% fetal bovine serum (FBS, Thermo-Fisher) and 1% penicillin/streptomycin (PS, Thermo-Fisher), and SAS was cultured in Dulbecco’s modified Eagle’s medium (DMEM, Thermo-Fisher) supplemented with 5% FBS and 1% PS. BEAS-2B was cultured in DMEM supplemented with 10% FBS and 1% PS. The cells were incubated in CO_2_ incubator (Thermo-Fisher). Incubation conditions were set as 37°C and the atmosphere at 5% CO_2_. The medium was replaced every 2 days. As 80–90% confluence was reached, cells were detached by 0.25% trypsin/EDTA (Thermo-Fisher).

### MTT assay

The MTT (3-(4, 5-dimethylthiazol-2-yl)-2, 5-diphenyltetrazolium bromide, Thermo-Fisher, Waltham, USA) assay was used to analyze the viability of cells using the colorimetric method. The yellow tetrazolium salt reduced to a purple formazan was used to evaluate cell viability. 7×10^3^ SAS/OECM1 cells were seeded in 96-well plates and were incubated overnight to allow cell confluence. Various concentration of HCD (0.5, 1, 5, 10, 20, and 50 μM for OECM1 and SAS; 1, 5, 10, and 20 μM for BEAS-2B) and cisplatin (5, 10, 20, and 50 μM for OECM1 and SAS; 10, 20, and 50 μM for BEAS-2B) were added into the wells, respectively. The plates were incubated at 37°C for 24 h. Then, 20 μL of MTT was added and incubated at 37°C for 3–6 h. After incubation for 3–6 h, medium and MTT were removed, and formazan was solubilized using dimethyl sulfoxide, the absorbance of 570 nm was measured by EnSpire Alpha plate reader (PerkinElmer, Waltham, USA). The absorbance was positively correlated to the number of viable cells so that cell viability was represented as the percentage of absorbance between treated and untreated cells.

### Cell cycle analysis by flow cytometry

7×10^4^ OECM1 and SAS cells were seeded per well in 12-well plates and were incubated overnight for cell adherence. Various concentrations of HCD and cisplatin were added and incubated for 12 and 24 h, respectively. Next, cells were harvested by trypsin and fixed with 70% ethanol at -20°C at least for 1 h. The samples were washed in cold PBS twice, and then incubated with 1 mL (v/v) staining solution (20 μg/mL of propidium iodide, 0.1% Triton X-100, 0.2 mg/mL RNase) at 37°C for 30 min. Finally, cells were analyzed by the flow cytometer (CytomicsTM FC500, Beckman, Fullerton, USA). Data from 10^4^ cells were collected for each data file.

### Western blotting

7×10^4^ of OECM1 and SAS cells were seeded into 12-well plate, when the cells reached about 80% confluence. OECM1 and SAS cells were treated with HCD for 24 h, respectively. After incubation, media was removed and washed twice with PBS and then cells were homogenized using protein lysis solution (RIPA buffer). The samples were centrifuged at 12000 ×g at 4°C for 30 min and the supernatant was kept at -20°C until the assay. Interested proteins were separated using sodium dodecyl sulfate polyacrylamide gel electrophoresis (SDS-PAGE) and subsequently transferred to a PVDF (Millipore, Bedford, USA) membrane. The blots were blocked with 5% non-fat milk in TBST saline (20 mM Tris–HCl, pH 7.4, 137 mM NaCl, and 0.05% Tween-20) at room temperature for 1 h and then incubated with the appropriate primary antibody at 4°C overnight. After wash, the blots were incubated with peroxidase conjugated secondary antibody for 1 h. Then, the desired proteins were visualized by ECL reagents (GE Healthcare, Pittsburg, USA) for 1 min, and LAS-3000 image system (FUJIFILM, Tokyo, Japan) was performed to detect protein signals. GAPDH was taken as an internal control for normalization. Table 5 lists the primary antibody and secondary antibodies.

**Table 5:** Primary and secondary antibodies used in this study

Antibody	MW (kDa)	Dilution	Sources
mTOR	289	1:1000	Cell Signalling
PI3K-ClassIII	100	1:1000	Cell Signalling
AMPKa	62	1:1000	Cell Signalling
P62	62	1:1000	Cell Signalling
AKT	60	1:1000	Cell Signalling
Beclin-1	60	1:1000	Cell Signalling
Cyclin D	34	1:1000	Cell Signalling
LC3-I	16	1:1000	Cell Signalling
LC3-II	14	1:1000	Cell Signalling
GADPH	36	1:10000	Cell Signalling
anti-Rabbit (IgG)		1:5000	GeneTex
anti-Mouse (IgG)		1:10000	GE

### Animal model

Six-week-old BALB/c nu/nu male mice (n= 30) were purchased from the National Laboratory Animal Center (Nan-Kang, Taiwan) and kept at controlled environmental conditions at room temperature (22 ± 2°C) with the humidity (55 ± 10%). The 12 h light (0600 am–1800 pm) and 12 h dark cycle was maintained throughout the study. The animals were fed a commercial diet and provided water *ad libitum*. The animal experimental protocols were followed as per the “Guide for the Care and Use of Laboratory Animals” of National Dong-Hwa University approved by the National Dong-Hwa University Animal Ethics Committee. SAS cells (8×10^5^ cells in 0.2 mL of DMEM) were subcutaneously injected into the right dorsal flank of nu/nu mice (n = 6 per group). When tumor volume reached 0.6 cm^3^, they were randomly allotted into 3 groups: Control, Cisplatin (0.1 mg/kg B.wt), and HCD (2.0, 6.5, and 19.5 mg/kg B.wt). Mice were intraperitoneally treated with HCD and cisplatin once every two days for a total of seven treatments. The body weight of inoculated tumor cell nude mice was measured once every two days. Tumor growth was measured twice weekly until sacrifice. The tumor volume was calculated based on the formula: (Length × Width^2^)/2.

### Tissue and histopathology

Mice were sacrificed by CO_2_ anesthesia and the tumors were excised immediately for measuring the tumor weights. One part of the tumor was weighed and the tissues were fixed in 4% formalin for histological analysis. The remainder of the tissue was immediately removed and stored at -80°C. The fixed tissue was dehydrated with alcohol and wax infiltration with Toluene: Paraffin (1:1, v/v) for 1 h. Tissue was embedded for 30 min at 65°C. Paraffin tissue sections were surface transverse sliced by the slicer (MICROM HM-325, Thermo-Fisher) into 5 μm of thickness. 5 μm tissue sections were stained with hematoxylin and eosin stain (H & E) (Sigma-Aldrich, St. Louis, USA). The completion of tissue sections was mounted with a coverslip after staining. Images were acquired using a phase contrast microscope (Carl Zeiss AG, Oberkochen, Germany) connected to a camera (Canon 700 D, Tokyo, Japan).

### Statistical analysis

Data were expressed as mean ± SE of at least three or more independent experiments. The results were analyzed by one-way analysis of variance (ANOVA) followed by a Tukey’s test. Significant differences (*P* < 0.05) between the means of control and treatment were analyzed. All statistical procedures were performed with GraphPad Prism Ver 5.0 (GraphPad Software, La Jolla, USA).

## Abbreviations

HCD: 16-hydroxycleroda-3, 13-dine-15, 16-olide
OSCC: oral squamous cell carcinoma
MTT: 3-(4, 5-dimethylthiazol-2-yl)-2, 5-diphenyltetrazolium bromide
H&E: hematoxylin and eosin stain
mTOR: mammalian target of rapamycin
PI3K: phosphatidylinositol 3 kinase
AMPK: AMP activated protein kinase
ROS: reactive oxygen species
MSN: mesoporous silica nanoparticle
Cisplatin: (SP-4-2)- diamminedichloroplatinum (II)
FBS: fetal bovine serum
PS: penicillin/streptomycin
DMEM: Dulbecco’s modified Eagle’s medium
SDS-PAGE: sodium dodecyl sulfate polyacrylamide gel electrophoresis
ANOVA: analysis of variance
